# Dysregulation of Dicer1 in Beta Cells Impairs Islet Architecture and Glucose Metabolism

**DOI:** 10.1155/2012/470302

**Published:** 2012-09-06

**Authors:** Amitai D. Mandelbaum, Tal Melkman-Zehavi, Roni Oren, Sharon Kredo-Russo, Tomer Nir, Yuval Dor, Eran Hornstein

**Affiliations:** ^1^Department of Molecular Genetics, Weizmann Institute of Science, Rehovot 76100, Israel; ^2^Department of Developmental Biology and Cancer Research, The Institute for Medical Research Israel-Canada, The Hebrew University Hadassah Medical School, Jerusalem 91120, Israel

## Abstract

microRNAs (miRNAs) play important roles in pancreas development and in regulation of insulin expression in the adult. Here we show that loss of miRNAs activity in beta-cells during embryonic development results in lower beta-cell mass and in impaired glucose tolerance. Dicer1-null cells initially constitute a significant portion of the total beta-cell population. However, during postnatal development, Dicer1-null cells are depleted. Furthermore, wild-type beta cells are repopulating the islets in complex compensatory dynamics. Because loss of Dicer1 is also associated with changes in the distribution of membranous E-cadherin, we hypothesized that E-cadherin activity may play a role in beta cell survival or islet architecture. However, genetic loss of E-cadherin function does not impair islet architecture, suggesting that miRNAs likely function through other or redundant effectors in the endocrine pancreas.

## 1. Introduction

Genome-encoded miRNA create a regulatory layer that impacts gene expression posttranscriptionally (reviewed in [1]). miRNA are important for beta-cell differentiation and function, and specific miRNAs have been proposed to regulate beta-cell genes [2–7].

miRNA are subject to extensive processing, including digestion by Drosha in the nucleus [8] and by Dicer1 (MGI: 2177178) in the cytoplasm [9]. Deletion of Dicer1 in the early pancreatic lineage, using a Pdx1-Cre mouse line, results in inactivation of the entire miRNA pathway in the early pancreatic bud and causes pancreas agenesis, demonstrating that miRNA are important for pancreas organogenesis [10]. The adult pancreas is also susceptible to loss of Dicer1, as pancreas morphology is distorted in a Dicer1 hypomorph model [11]. Furthermore, we have recently shown that Dicer1 and miRNA function is critical for maintenance of the beta-cell hormone-producing phenotype, by maintaining the proper balance of transcriptional activators and repressors upstream of insulin expression [12]. 

E-Cadherin is a transmembrane protein encoded by the gene Cdh1 (MGI: 88354), which is involved in homotypic cell-cell interactions [13]. E-Cadherin function was suggested to play a role in endocrine cell clustering and in the establishment of normal islet morphology and function [14–18].

In this work we show the importance of Dicer1 for beta-cell survival and islet architecture. Dicer1-null beta cells are progressively lost within the first few weeks after birth. However, wild-type beta cells, which do not undergo recombination, repopulate the islet. Dicer1-null beta cells also exhibit changes in the distribution of E-Cadherin, reminiscent of previous reports (e.g., [16]). However, genetic loss of Cdh1, which encodes for E-Cadherin, did not exhibit detectable glycemic or tissue phenotype.

## 2. Results

### 2.1. Beta-Cell-Specific Disruption of Dicer1 During Embryonic Development Causes Juvenile Glucose Intolerance

**Figure 1:**
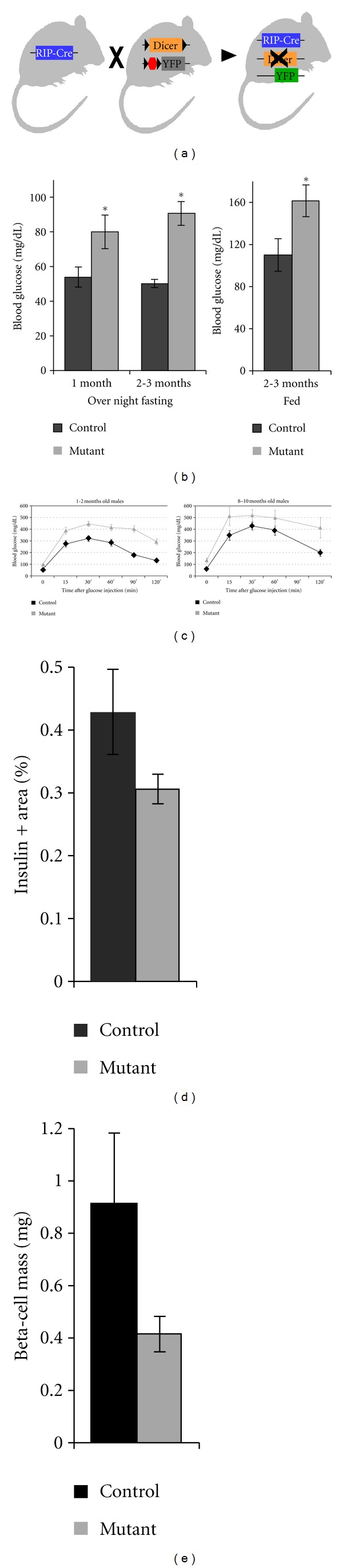
Dicer1 conditional knockout in beta cells, results in impaired glucose tolerance and reduction in beta-cell mass. A diagram of mouse genetics for generation of RIP-Cre; Dicer1^LoxP/LoxP^ mutants that additionally harbor Rosa-EYFP reporter (a). Blood glucose levels of RIP-Cre; Dicer1^LoxP/LoxP^ mutants and RIP-Cre; Dicer1^LoxP/+^ controls, after overnight fasting or when randomly fed (b), and during standard glucose tolerance test (GTT) at multiple time-points after intra-peritoneal injection of a glucose bolus (c). Assessment of the percentage of insulin-positive sectional area relative to the total pancreas area in serial sections of mutant and control pancreata (d)). Calculated beta-cell mass, corrected to pancreas weight (e). *P < 0.05.

Loss of Dicer1 function blocks the maturation of miRNA species, thus providing a platform for assessment of the overall contribution of miRNAs to beta-cell function *in vivo*. With this goal in mind, we created RIP-Cre, Dicer1^LoxP/LoxP^ mice, in which Dicer1 conditional allele is inactivated by Cre thereby preventing miRNA processing. This strain was created by crossing a Dicer1 conditional allele [19] onto a conditional Cre transgene, driven by the rat insulin promoter [20]. Concomitant with Dicer1 inactivation, Cre recombinase activated an enhanced yellow fluorescent protein (YFP) reporter, integrated into the ROSA26 locus [21]. Thus, cells in which recombination occurred lost Dicer1 activity and were labeled with YFP. Control mice were heterozygous for the Dicer1 allele (RIP-Cre; Dicer1^LoxP/+^) and harbored YFP reporter (Figure 1(a)). RIP-Cre; Dicer1^LoxP/LoxP^ animals developed fasting and fed hyperglycemia (Figure 1(b)). Accordingly, when RIP-Cre; Dicer1^LoxP/LoxP^ animals were tested for glucose clearance after intraperitoneal injection of 2 mg/gr glucose they exhibited impaired glucose tolerance at the age of 1-2 months, which was evident also at the age of 8–10 moths (Figure 1(c)).

### 2.2. Reduced Beta-Cell Mass in Dicer1 Mutants

In order to understand the cause for impaired glucose tolerance, we performed morphometric analysis of RIP-Cre; Dicer1^LoxP/LoxP^ pancreata. Beta-cell mass was calculated from the total insulin-positive immunoreactivity area, relative to the total pancreas area and weight (see methods). Our analysis, performed at the age of 4 months, indicates that RIP-Cre; Dicer1^LoxP/LoxP^ mutant beta-cell mass is reduced compared to control (Figures 1(d) and 1(e)). However, the compromised functionality of the pancreas likely results also from impaired insulin synthesis and defective exocytosis, as these processes were previously shown to be controlled by miRNAs.

**Figure 2:**
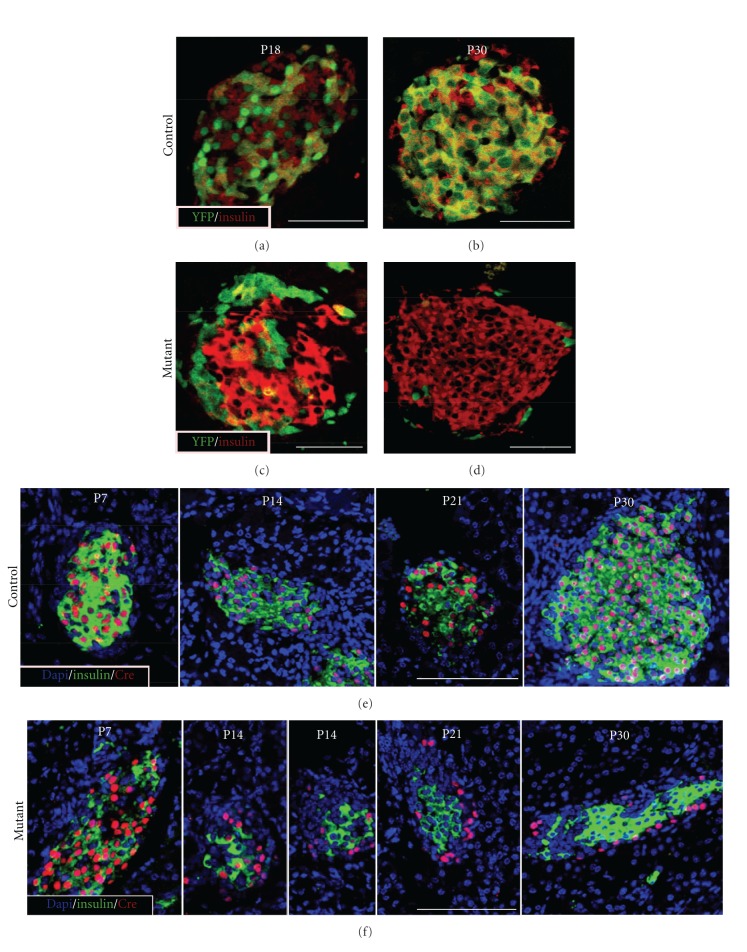
Progressive disappearance of Dicer1 mutant cells during post-embryonic growth of islets of Langerhans. YFP-positive (green) cells are accompanied by wild-type beta cells, which do not express YFP and are immunostained for insulin (red). These two populations of beta cells are comparably abundant in control RIP-Cre; Dicer1^LoxP/+^ islets, at postnatal days 18 (a, P18) and 30 (b, P30). However, “mutant” beta cells in islets of RIP-Cre; Dicer1^LoxP/LoxP^ animals that are detected at P18, are progressively lost and are rarely detected at P30 (c, d). Cre-positive ‘control' cells are dispersed throughout islet tissue of Cre; Dicer1^LoxP/+^ at multiple postnatal time points (e). Cre Immunofluorescence reveals the progressive loss of Cre-positive beta-cell from RIP-Cre; Dicer1^LoxP/LoxP^ islets, that are depicted only at the islet periphery at P21 and P30 (f). Bar −50 *μ*m.

To evaluate insulin expression and synthesis, we performed immunoflouresent analysis. Beta cells, that expressed YFP, in which Dicer1 activity was disrupted, did not express insulin (Figures 2(a)–2(d)), suggesting that insulin synthesis is inhibited in Dicer1 knockout beta cells. Nonetheless, cells that lost Dicer1 activity did not express alternative endocrine markers, namely, glucagon and somatostatin (Figure S1). Noteworthy, wild-type beta cells, which did not undergo recombination, within mosaic mutant islets, did not express YFP and maintained the expression of insulin (Figures 2(a)–2(d)). These observations are consistent with our previous study of insulin synthesis in Dicer1 knock-out beta cells in adulthood [12].

### 2.3. Dicer1 Mutant Cells Are Progressively Lost from Mutant Islets

In early postnatal life, Dicer1 knock-out beta cells were dispersed uniformly throughout the islet tissue. However, the abundance of Dicer1-null cells progressively decreased. Thus, only a minority of the cells were YFP-positive (and presumably Dicer1-negative) at the age of one month (Figures 2(b) and 2(d)). 

To rule out a potential artifact, related to the activity of the Rosa-YFP reporter in our model, we sought to detect directly the expression of the Cre transgenic protein, which is controlled by the activity of rat minimal insulin promoter in beta cells. Immunofluorescence studies of pancreata revealed that, at P7, Cre-expressing cells were evenly spread within islets. Examination of older animals revealed a gradual loss of Cre-expressing cells in mutant animals ending at P30, when almost no Cre-positive cells were detected. Furthermore, it appears that a second population of seemingly-normal beta cells, which did not express Cre and maintained insulin expression, gradually compensated for the reduction in Cre-positive cells. Intriguingly, the remaining Cre-expressing cells were progressively and preferentially found at the periphery of the islet (Figure 2(f)). In control islets, however, Cre expressing cells were abundant at all studied time-points and show unaltered distribution (Figure 2(e)).

### 2.4. Apoptosis Is Not Detected in Dicer1 Mutant Beta Cells

One plausible explanation for the loss of Cre-positive cells may be related to apoptosis, which has been described in some Dicer1 models. However, other cell types do not seem to respond to loss of Dicer1 activity by a programmed cell death response (e.g., [22–24]). In order to evaluate potential apoptosis, we immunostained pancreas sections for activated caspase3 at different time points. Surprisingly, we did not detect apoptosis (Figure S2) in screening 3500 beta cells in 10 different animals by two independent investigators. A third qualitative evaluation of pyknotic nuclei on hematoxylin/eosin staining by a pathologist was also negative (not shown). We conclude that RIP-Cre; Dicer1^LoxP/LoxP^ beta cells do not undergo observable apoptosis. While this study is rather exhaustive and consistent with the work of Kalis et al., [25], we cannot exclude, for example, high clearance dynamics or other reasons for false-negative detection of apoptosis in dying beta cells. 

### 2.5. Abnormal E-Cadherin Expression in Dicer1-Null Cells

Since we noticed that mutant beta cells tend to be positioned at the periphery of the islets (Figure 2), we hypothesized that these cells might have altered adhesion properties. We therefore quantified the expression pattern of E-Cadherin along the beta-cell membrane, based on qualitative observations of Yamagata et al., [16]. 

This analysis was unbiased, taking a blinded approach. Thus, we first gave each beta-cell a binary value for “normal” or “abnormal” E-cadherin expression and only then, in a secondary step, we have annotated cells as either mutant (YFP-positive) or control (YFP-negative). Then the percentage of cells with abnormal E-Cadherin staining was calculated from the total counted cells.

**Figure 3:**
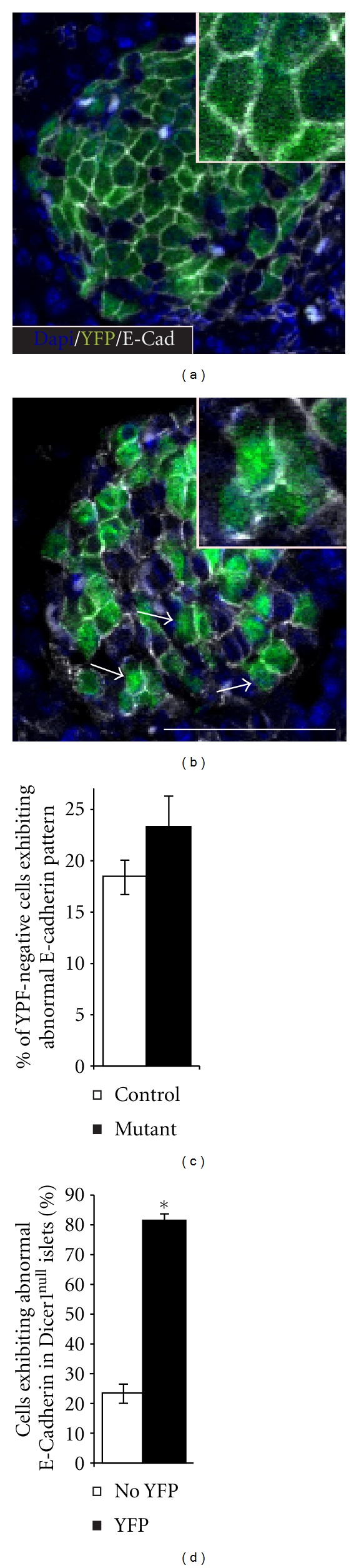
Dysregulated membranal E-Cadherin expression in Dicer1 mutant cells. Representative Control (RIP-Cre; Dicer1^LoxP/+^, (a)) and Mutant (RIP-Cre; Dicer1^LoxP/LoxP^, (b)) P11 islets, immunostained for E-Cadherin (white), and YFP (green) were taken for quantitative analysis. Magnified field in inset. Comparable E-Cadherin expression in YFP-negative cells in Mutant and Control animals (c). The incidence of Dicer1 mutant beta cells exhibiting dysregulated E-Cadherin expression is higher than in control beta cells, which do not express YFP, in the same islet (d). Bar −50 *μ*m. *P < 0.01.

Initially, we assessed baseline E-cadherin continuity in control beta cells, which did not undergo recombination by comparing the YFP-negative cells in mutant and control pancreata (genotypes: RIP-Cre; Dicer1^LoxP/LoxP^ and RIP-Cre; Dicer1^LoxP/+^, resp. Figures 3(a) and 3(c)). This analysis suggested a baseline value denoting ~18% of cells as harboring abnormal membranous E-Cadherin pattern. This value is probably sensitive to staining and microscopy biases and is subjective in nature. However, this evaluation method was used in a blinded fashion for controls and mutants and therefore provides the grounds for comparing E-Cadherin expression in mutant cells. 

This approach revealed comparable membranous E-cad pattern in control cells of mutant and wild-type animals (Figures 3(a) and 3(c)). However, using the same method for P11 and P18 islets of RIP-Cre; Dicer1^LoxP/LoxP^, we uncovered significant downregulation of E-cadherin continuity in YFP-positive/Dicer1 mutant cells (Figures 3(b) and 3(d)). Thus aberrant distribution of E-Cadherin at the beta-cell membrane was more abundant in beta cells that lost Dicer1 activity.

### 2.6. Loss of Cdh1 Expression Affects Neither Glucose Homeostasis Nor Islet Architecture

**Figure 4:**
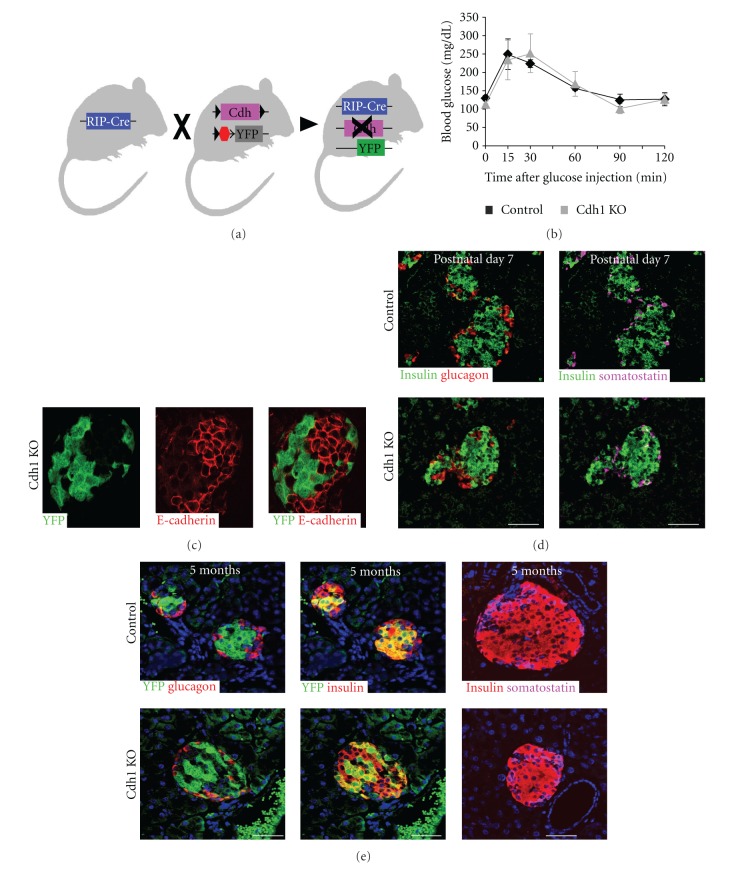
Loss of Cdh1, encoding for E-cadherin, does not attenuate glucose homeostasis or islet architecture. A diagram revealing mouse genetics steps for loss of Cdh1 in beta cells (a). RIP-Cre; Cdh1^LoxP/LoxP^ mice, marked as Cdh1 KO, tolerate at the age of five months intra-peritoneal glucose bolus, like control counterparts (b). RIP-Cre; Cdh1^LoxP/LoxP^ mutant cells, which do not express E-Cadherin and are marked by the expression of a YFP reporter, exhibit normal cellular morphology within mature islets, at the age of five months (c). Endocrine markers are comparably expressed in mutant and control islets at the age of 7 days, (d) and of five months, (e). Bar −50 *μ*m.

To investigate the hypothesis that E-Cadherin may be involved in islet biology, we took a mouse-genetics approach. We created RIP-Cre; Cdh1^LoxP/LoxP^ mouse line for specific loss of E-Cadherin expression in beta cells, by crossing a Cdh1 conditional allele [26] to an RIP-Cre transgene [20] (Figure 4(a)). Unexpectedly, adult RIP-Cre; Cdh1^LoxP/LoxP^ mice exhibited normal glucose tolerance (Figure 4(b)). Analysis of pancreata from RIP-CRE; Cdh1^LoxP/LoxP^ that also harbor an inducible YFP transgene (i.e., recombined cells lost E-Cadherin expression and were labeled with YFP) by coimmunodetection of E-cad and of YFP showed that tissue composition is unaffected by loss of E-cad (Figure 4(c)). Thus, surprisingly, wild-type and E-Cadherin-null beta cells reside in the same islet. Furthermore, RIP-Cre; Cdh1^LoxP/LoxP^ islet morphology was normal between P7 and the age of five months (Figures 4(d) and 4(e)). From this genetic study we conclude that loss of E-cadherin is not sufficient to interrupt any observable cellular features or endocrine physiology, *in vivo*. Furthermore, although E-cadherin distribution is impaired in Dicer1 mutant beta cells, direct knockout of E-Cadherin in the same cell population did not result in similar phenotypes. We therefore conclude that other elements, downstream of Dicer1 affect islet architecture and the loss of beta cells. However, E-Cadherin may be a marker of attenuated adhesion in Dicer1 mutant beta cells and it may function redundantly with some other factors in the upkeep of beta-cell epithelial features.

## 3. Discussion

Our results demonstrate the importance of Dicer1 in beta-cell survival and are also consistent with the function of miRNA in maintenance of insulin expression in the adult islet [12]. While this paper was in preparation, Kalis et al. reported a similar model [25]. Together the works of Kalis et al. and ours provide a consistent view of the importance of Dicer1 and miRNAs for juvenile glucose homeostasis. Dicer1 is essential for beta-cell survival. Progressive loss of beta cells in the studies of Kalis et al. [25] and in our study was not depicted by standard apoptotic markers, which might be related to limited assay sensitivity or to nonapoptotic cell death. In addition, mutant beta cells are probably diluted upon the proliferation of wild-type cells in chimeric islets. 

When Dicer1is deleted in adulthood, islet architecture stays intact[12]. This may reveal a role for Dicer1 during the embryonic or early postnatal period, as previously suggested [25]. However, another possibility is that changes in tissue composition are more readily evident in growing islets of the newborn than in older, less dynamic, adult tissue. If this is true, then similar changes in islet architecture may be potentially observed also in the adult model, over longer periods of time. 

The positioning of mutant beta cells at islet periphery after postnatal day 14 suggests that differential adhesion properties may preferentially encourage homotypic adhesion between wild-type cells. However, rather unexpectedly, direct knockout of E-Cadherin in beta cells did not reveal any physiological phenotype, neither was it associated with changes in islet morphology, or the expression of endocrine markers. This is surprising since past reports suggested a role for E-Cadherin in clustering and in function of endocrine cells [14–18, 27]. Therefore, our *in vivo* results point to the existence of alternative or redundant molecular mechanisms for controlling beta-cell adhesion and islet epithelial properties.

The RIP-Cre; Dicer1^LoxP/LoxP^ model exhibits chimerism denoted by the presence of both mutant and wild-type beta cells in the same islet. Detailed analysis of a temporal series of mutant pancreata revealed that a wild-type population is replacing Dicer1-null beta cells and eventually repopulates the whole islet. Interestingly, reminiscent tissue dynamics are observed in conditional knock-out model of the insulin receptor substrate 2 (Irs2) gene. In that model, a subset of the beta cells, which evaded Cre-dependent recombination, repopulated the endocrine pancreas [28]. Therefore, extensive compensatory growth of wild-type beta-cell clones may reflect a physiological response to impaired endocrine function, which is imposed by loss of genetic function in subsets of the cells in the organ. This may be observed in other conditional knock-out models, which exhibit chimeric and incomplete recombination, regardless of the preceding genetic insult. Our observations suggest that wild-type clone proliferation capacity is nonetheless limited. Thus, RIP-Cre; Dicer1^LoxP/LoxP^ mice manifest impaired glucose tolerance at the age of two months but also at the late age of 9 ± 1 month, long after Dicer1-null beta cells become an insignificant minority within the organ. This is consistent with the reported finite potential for compensatory proliferation of beta cells and their progenitors [29], even if the required beta-cell mass for euglycemia is not met. 

In summary, our study reveals Dicer1 importance for beta-cell survival and the normal function of the insulin axis. The unexpected islet dynamics suggest that Dicer1 mutant cells are outcompeted in time by wild-type beta cells that repopulate the islet, providing an intriguing model that uncovers the limitations of compensatory proliferation in meeting the physiological needs of the animal.

## 4. Materials and Methods

### 4.1. Mouse Handling and Physiology

The following mouse alleles were studied: rat insulin promoter-Cre transgene [20], Dicer1^flox^ allele [19], R26R-EYFP [21], and Cdh1 [26]. Mice were housed and handled in accordance with protocols approved by the Institutional Animal Care and Use Committee of WIS. Glucose tolerance tests were performed by intra-peritoneal injection of glucose (2 mg/g BW), after an overnight fast and measuring blood glucose levels using an “*Ascensia elite*” glucometer. Primers for PCR genotyping are listed in Supplementary materials available online at doi: 10.1155/2012/470302.

### 4.2. Pancreatic Histology and Immunohistochemistry 

Dissected pancreata were fixed in 4% paraformaldehyde, at room temperature for 2 h (P7), 3.5 h (P14, P21), or 24 h (P30) and then processed into paraffin blocks. Antigen retrieval in a 2100-Retriever (PickCell Laboratories, The Netherlands) was performed on 5 *μ*m thick rehydrated sections prior to immunostaining with antibodies described in the Supplementary Material. Fluorescence images were captured using a Zeiss LSM510 and LSM710 Laser Scanning confocal microscope system under a magnification of ×40. Nuclei counter-stained with Hoechst, 1 *μ*g/mL (Sigma). Beta-cell mass was determined by analysis of consecutive paraffin sections 75 *μ*m apart spanning the entire pancreas (approximately 20 sections/pancreas), stained for insulin and hematoxylin. Digital images of sections at a magnification of ×40 were obtained and stitched using NIS-Elements software (NIKON), and the fraction of tissue covered by insulin staining was determined. The mass of beta cells was calculated as the product of pancreas weight and the fraction of tissue covered by beta cells. Detailed beta-cell mass protocol is described in Nir et al. [30].

### 4.3. Statistical Analysis

All statistical analyses were performed using Student's *t*-test and ANOVA as needed and are displayed as mean ± s.e.m. of three or more samples/experiments.

## Supplementary Material

Supplementary materials for Mandelbaum et al., includes Sup. Figure S1 “Dicer1 null cells do not express alternative endocrine cell marker” and Figure S2 “Dicer1 null cells do not undergo apoptosis” and a table of primers used for this study.
