# Supplementary material for: Dysregulation of Dicer1 in Beta Cells Impairs Islet Architecture and Glucose Metabolism
Source: Exp Diabetes Res. 2012 Sep 6;2012:470302. doi: 10.1155/2012/470302 (PMC3443614; doi:10.1155/2012/470302)
Supplement: Supplementary file 1 — Supplementary materials for Mandelbaum et al., includes Sup. Figure S1 “Dicer1 null cells do not express alternative endocrine cell marker” and Figure S2 “Dicer1 null cells do not undergo apoptosis” and a table of primers used for this study. [file 470302.f1.pdf]

Supplementary

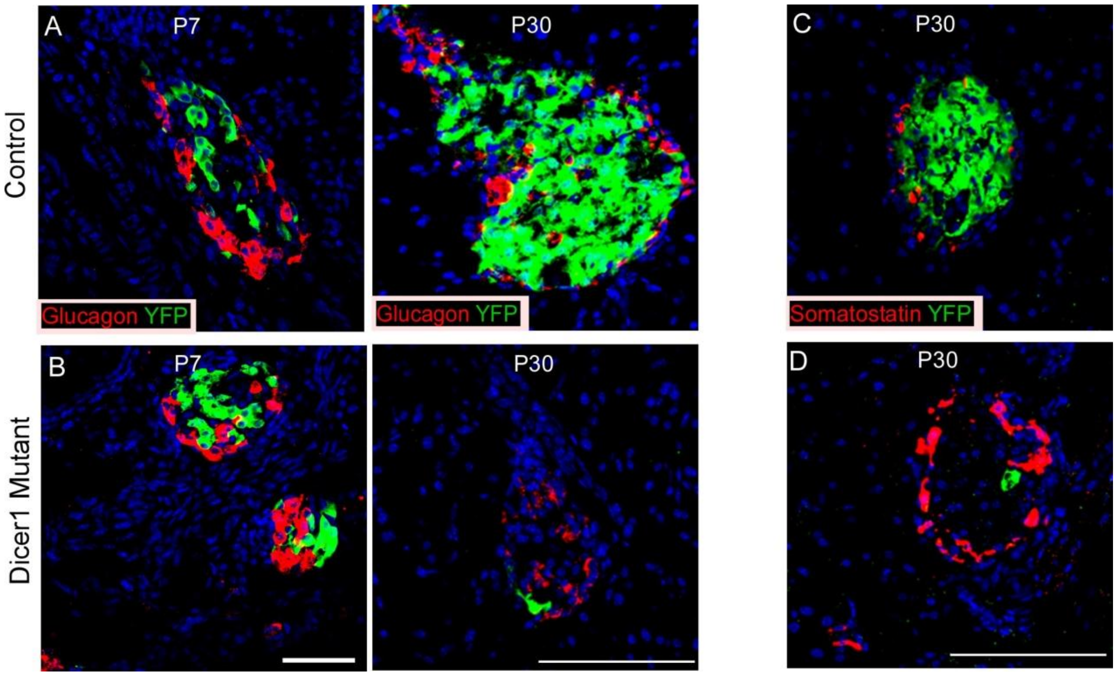

Figure S1: Dicer1 null cells do not express alternative endocrine cell marker. Normal glucagon expression in alpha cells of RIP-Cre;Dicer1$^{LoxP/LoxP}$ islets and control at post-natal days 7 and 30 (A,B) and of somatostatin at post-natal day 30 (C,D). Notably, YFP-positive Dicer1-null cells are rarely seen at the age of 30 days, consistent with figures in the main text. Bar - 50µm.

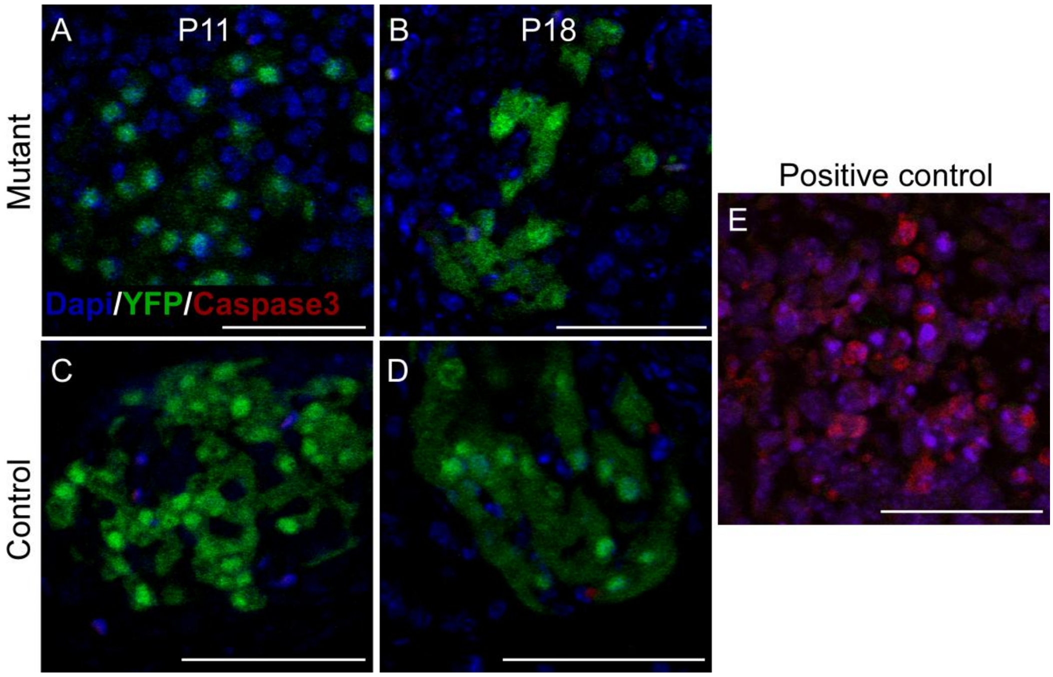

Figure S2: Dicer1 null cells do not undergo apoptosis. Fluorescenct immunostaining for activated-caspase 3, neither reveal apoptosis in Dicer1 null cells (A,B) nor in control pancreata (C,D). Positive signal, providing technical control for detection of activated-caspase 3 in irrelevant tissue (embryonic branchial arches, E) Bar - 50µm.

Primers table

Gene name (F-forward, R-reverse)	Forward Primer
Dicer1_F	CCTGACAGTGACGGTCCAAAG
Dicer1_R	CATGACTCTTCAACTCAAACT
Cre_F	TGCCACGACCAAGTGACAGC
Cre_R	CCAGGTTACGGATATAGTTCATG
Rosa26YFP_F	TGTTTTGGAGGCAGGAAGCACTTG
Rosa26YFP_R Wild-type	GCTGCATAAACCCCAGATGACTCC
Rosa26YFP_R knockin	CATCAAGGAAACCCTGGACTACTG
Cdh1_F	GGG TCTCAC CGT AGT CCT CA
Cdh1_R	GAT CTT TGG GAG AGC AGT CG
